# Effectiveness of iodine for continuous decontamination of dental unit waterline

**DOI:** 10.1038/s41405-023-00160-w

**Published:** 2023-07-22

**Authors:** Chatchaya Petchphayaprai, Chutimon Chotipan, Pitchayapa Sa-ngiampak, Kittisak Thotsaporn, Ruchanee Salingcarnboriboon Ampornaramveth

**Affiliations:** 1 Center of Excellence on Oral Microbiology and Immunology, Department of Microbiology, Faculty of Dentistry, Chulalongkorn University, Bangkok, Thailand; 2 Department of Biochemistry, Faculty of Dentistry, Chulalongkorn University, Bangkok, Thailand

**Keywords:** Dentistry, Occupational health

## Abstract

**Objective:**

Dental Unit Waterlines (DUWLs) are contaminated by various species of microorganisms. DUWLs should be disinfected appropriately to control microbial contamination. This study investigated the effectiveness of devices continuously releasing iodine to control microbial contamination in DUWLs.

**Materials and methods:**

Ten dental chair units (DCU) at Chulalongkorn University were randomized into the iodine and control groups. After setting iodine treatment devices, the DCU was allowed to operate normally. 25 ml of water from airotors lines were collected weekly for enumerating bacteria. The viability of biofilms in DUWLs was quantified by ATP testing kit. The amount of iodine released into the procedural water was also quantified.

**Results:**

The continuous presence of iodine could significantly control bacterial contamination in the DUWL to be less than 500 CFU/mL, the standard level recommended by the Centre for Disease Control and Prevention (CDC). Iodine treatment can reduce bacterial CFU up to 98–100%. Biofilm viability in the iodine group was slightly lower than that of the control group though not statistically significant. After eleven months, the average iodine release was measured to be 3.6 ppm which is still effective in controlling bacterial contamination.

**Conclusion:**

Continuously supplying iodine in DUWLs effectively controls microbial contamination.

## Introduction

The critical component of dental chair units is dental unit waterlines (DUWLs). Dental unit waterlines serve as pipelines to deliver fresh water for cooling and irrigating during dental procedures. Waterlines are made of silicone rubber or polymer tubes. This pipeline is always filled with water, creating an environment suitable for biofilm formation. Once formed in the waterlines, biofilms are extremely difficult to eliminate [1, 2]. These biofilms will therefore release planktonic microorganisms into the procedural water of the dental chair unit. Due to the aquatic environment in the pipeline, contamination in DUWLs is unavoidable [3, 4]. According to American Dental Association (ADA) standard, the prevalence of bacterial contamination of DUWLs was estimated to be as high as 85.0%. While the prevalence of pathogenic species such as *Legionella pneumophila* and *Pseudomonas aeruginosa* is 12.0% and 8.0%, respectively [5]. DUWLs should be cleaned and disinfected appropriately to reduce biofilm and microbial contamination as low as possible, to meet the standard of drinkable water [2, 5, 6].

Various microorganisms, including bacteria, fungi, viruses, and protozoa, contaminate DUWLs [7]. The most common form of microorganisms found in DUWL is gram-negative bacteria, some of which are opportunistic pathogens [7, 8]. These pathogens harm not only vulnerable groups of patients, such as immunocompromised and the elderly, but also the dental staff in the clinic [2, 6].

The Centre for Disease Control and Prevention (CDC) recommends that procedural water for nonsurgical dental procedures contain less than 500 CFU/ml of heterotrophic water bacteria [9, 10]. There are many methods to reduce the contamination of DUWLs, including non-chemical and chemical approaches [11, 12].

Treatment by chemical agents can be performed as intermittent or continuous methods. According to previous reports, continuous chemical treatment is more effective in reducing bacterial contamination and controlling biofilm formation in the DUWLs [13, 14]. Several products have been introduced for continuous chemical treatment methods, such as chlorine dioxide, hydrogen peroxide, chlorhexidine gluconate, and iodine [11, 12, 15]. Iodine is a potent oxidizing agent that can kill microorganisms such as bacteria by attaching to microbial plasma membranes and inhibiting protein function. The principal mechanism of oxidizing agents in killing microorganisms is to disrupt cellular functions and reduce viability [16]. Iodine has been used for many purposes, such as wound antiseptic, water disinfection, and preventing goiter by adding it to drinking water. There are many forms of iodine, including organic iodide compounds such as bis-glycinate hydroiodide, potassium tetraglycine triiodide, iodophors (Iodine with solubilizing compounds), and other iodine release systems such as iodine-incorporated resins. DentaPure independent water bottle cartridge DPID365B, a commercially available continuous iodine treatment system, releases a low dose of iodine to decontaminate DUWLs. Despite being widely used worldwide, the effectiveness of this device in real clinical settings has not been reported elsewhere. This study aims to investigate the efficacy of iodine-releasing cartridges in controlling bacterial contamination and biofilms in DUWLs from ten similar dental chair units at Chulalongkorn University.

## Materials and methods

### Sample collection

The sample-sized calculation was performed by G Power 3.1 software. Using the effect size of 2.67, calculated from a previous similar study [17]. With a significance criterion of α = 0.05 and power = 0.95, the minimum sample size needed with this effect size is *N* = 4. Ten similar dental chair units of the same model and use life at the faculty of Dentistry Chulalongkorn University were randomly selected. The units were divided into two groups: five units of the control group with no intervention added to the waterlines and five units of iodine treated group in which the waterlines were continuously disinfected by installing iodine-releasing cartridge systems (DentaPure™ DP365B Independent Water Bottle Cartridge, HuFriedyGroup, Chicago, USA). The system contained non-allergenic iodinated resin beads, which released 2–6 ppm of atomic isotopes of elemental Iodine (I_2_) during a typical dental treatment. During the experimental period, the dental chair units normally operated at official working hours, 5 days a week.

Sample collection was performed every Wednesday, in the middle of the week, to avoid the variability in the data from stagnant water during the holiday. 25 ml of water samples were collected from the airotor lines of each dental unit after flushing the pipe for 1 min. As baseline water contamination, the samples were collected 1 month before installing iodine water treatment cartridge systems. Then the samples were collected every Wednesday continuously for nine weeks.

The Ethics Committee has approved the protocols of this study (HREC-DCU 2021-028) of the Faculty of Dentistry, Chulalongkorn University, Thailand, to be carried out and/or amended as follows in compliance with the ICH/GCP.

### Enumeration of bacterial contamination in DUWLs

The water samples were sonicated for 10 s to disperse the cluster of microorganisms. Serial tenfold dilution was performed, and 100 μl of the samples were plated onto R2A agar plates. All plates were incubated at 35 °C for 3–5 days then bacterial colonies were counted. The numbers of colonies were converted into colonies forming units per mL (CFU/mL). The number of bacteria in each dental chair unit at each time point was compared to the initial amount at baseline, calculating the percent CFU reduction from the baseline data. The log CFU reduction was calculated by taking log[*A*/*B*] (*A* = the average amount of bacteria each week, *B* = the number of bacteria at baseline).

### Biofilm viability assessment by measurement of adenosine triphosphate

At the end of the experiment (at week 9), the DUWLs in the path that delivers water to the airotor line inside the control box of dental chair units were sectioned into 5 mm lengths and kept in 0.9% sterile normal saline solution. The procedure was repeated at the same position in every dental chair unit of the control and iodine group. The lines were split in half. The biofilms were swabbed completely from a 5 mm-length of duct to remove all biofilms and measured the amount of adenosine triphosphate (ATP) by ATP testing kits (3 M™ Clean-Trace™ UXL100, Maplewood, USA). These kits can detect the presence of microbial contamination in DUWLs. The amount of ATP in relative light units (RLU) represents the relative bacterial vital activities.

### Measure iodine concentration

After installing the iodine treatment cartridge for 11 months, the iodine concentration in water samples was determined by an iodide electrode (Istek Inc, Korea) and benchtop pH meter (Orion Star™ A111 Benchtop pH Meter). The electrode measures iodide ions represented by water electric potential in mV. Then it converts electric potential to iodine concentration by comparing it to the standard iodine solution at 1, 2, 2.5, and 5 ppm. The water samples were collected again to measure bacterial contamination that represented the long-term effectiveness of the iodine cartridge.

### Statistical analysis

Statistical analysis was performed using the Shapiro-Wilk test for normality of data distribution testing. The independent T-test from the SPSS program was used for data analysis. The *p* value ≤ 0.05 confidence interval was considered significant.

## Results

**Fig. 1:**
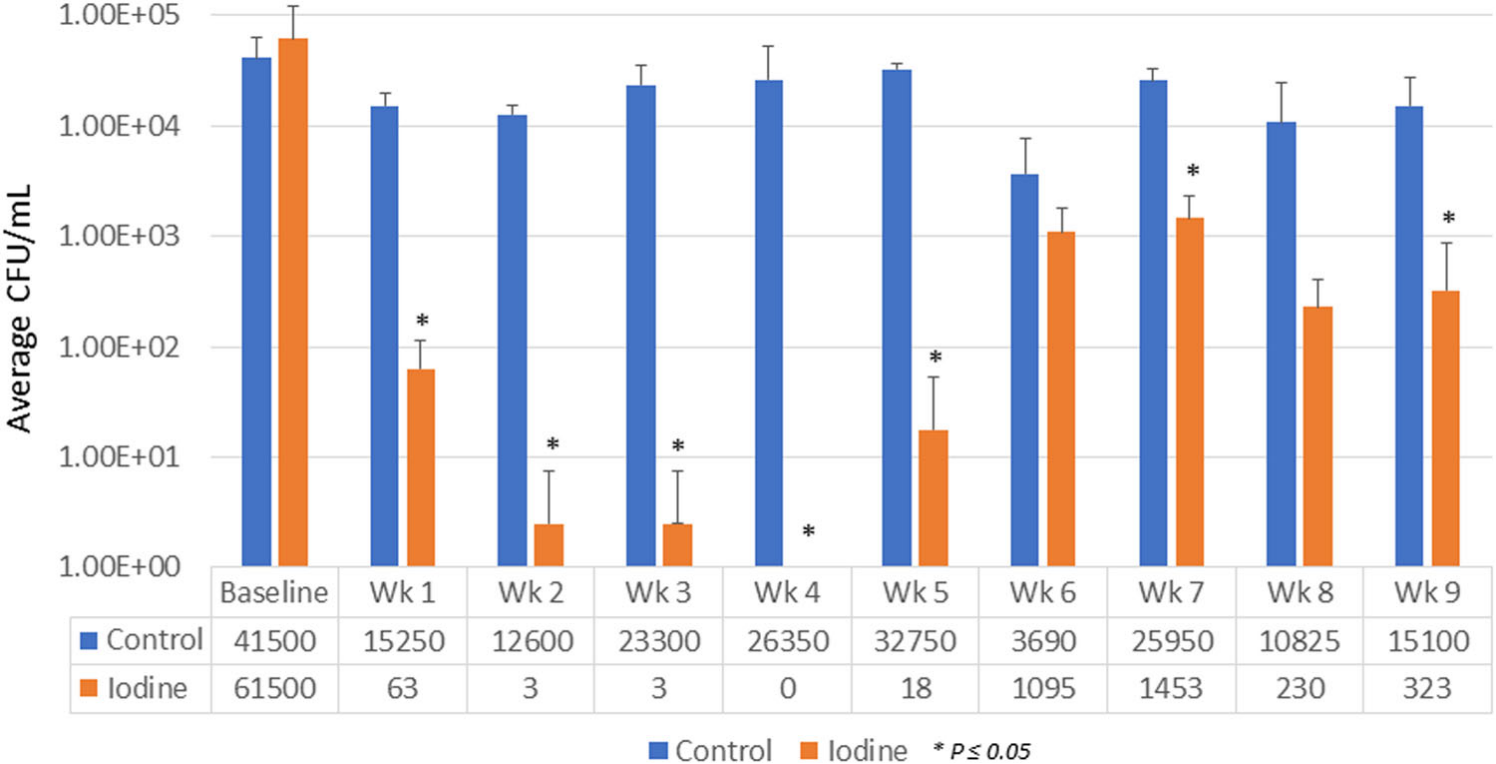
DUWLs bacterial contamination. Bacterial numbers recovered from the airotor lines of dental chair units were represented in average CFU/mL. The table below presents the average number of bacterial counts at each time point. The blue bars indicate the average CFU/ml in dental unit output water from the control group. The orange bars indicate the average CFU/ml in dental unit output water from the iodine group.

**Fig. 2:**
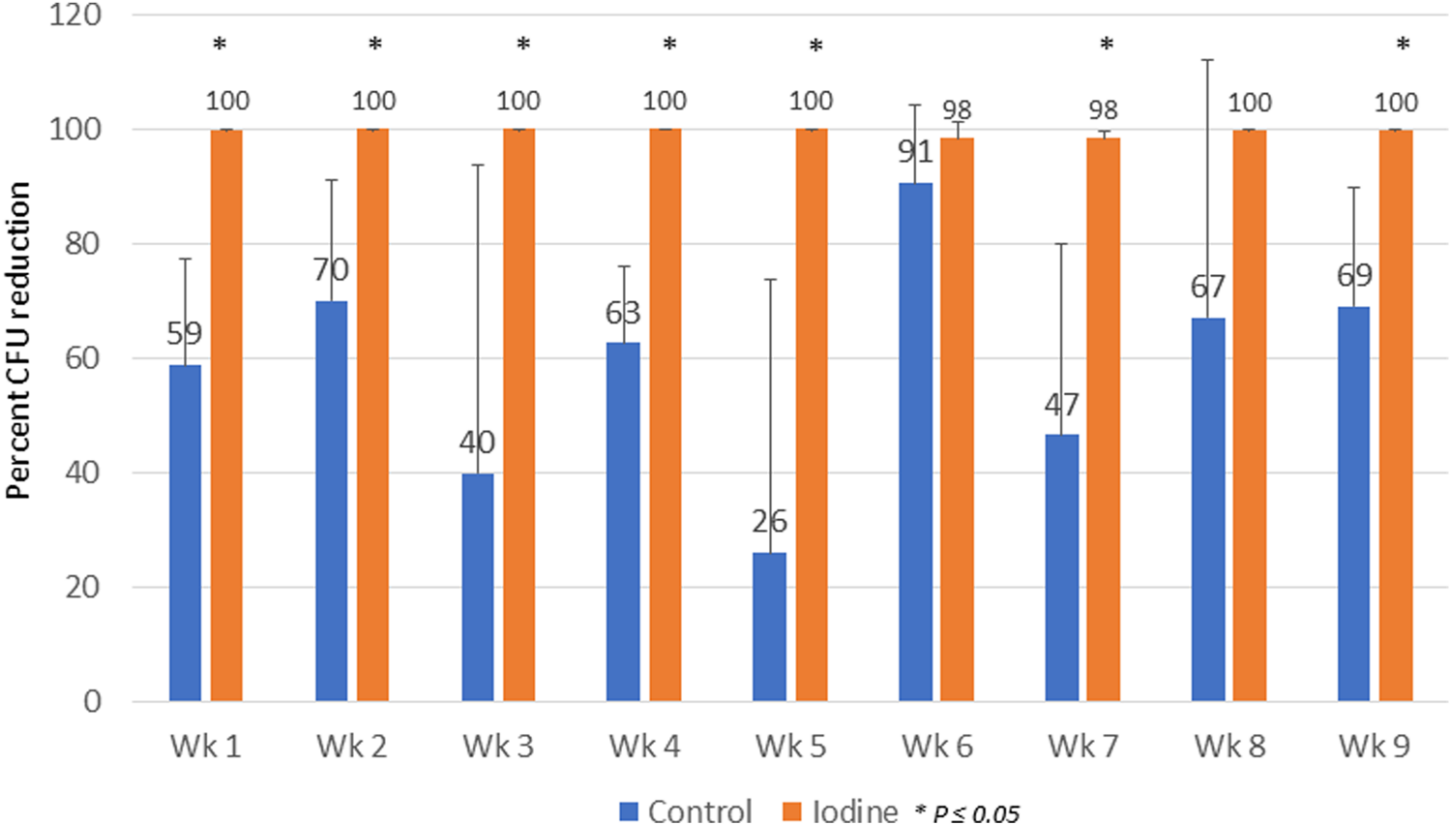
Percent CFU reduction. Percent CFU reduction demonstrated the percentage difference between CFU/ml during the experimental period compared with the baseline in the same DCU. The blue bars indicate the percent CFU reduction in the control group. The orange bars indicate the average percent CFU reduction in the iodine group. The chart below also presents the average percent CFU reduction at each time point. *Indicate statistically significant difference at *p* ≤ 0.05.

Dental unit waterlines (DUWLs) of ten dental chair units were highly contaminated with bacteria, with an average of 41,500 ± 21,016 and 61,500 ± 61,005 CFUs/ml) in the control and iodine groups, respectively. During the experimental period, the highest average CFUs/ml of all DCUs in the control group was 32,750 ± 3594 CFUs/ml (at week 5) compared to 1452 ± 854 CFUs/ml in the iodine group (at week 7) (Fig. 1). The bacterial count in the iodine group was lower than 500 CFU/ml in almost all weeks, except week 6 and 7, which meet the standard of water contamination recommended by the US CDC for nonsurgical dental procedures (Fig. 1). There were statistically significant differences in the bacterial count from DUWLs of the iodine and control groups. The percent CFU reduction in the iodine group ranges from 98 to 100% (Fig. 2).

**Fig. 3:**
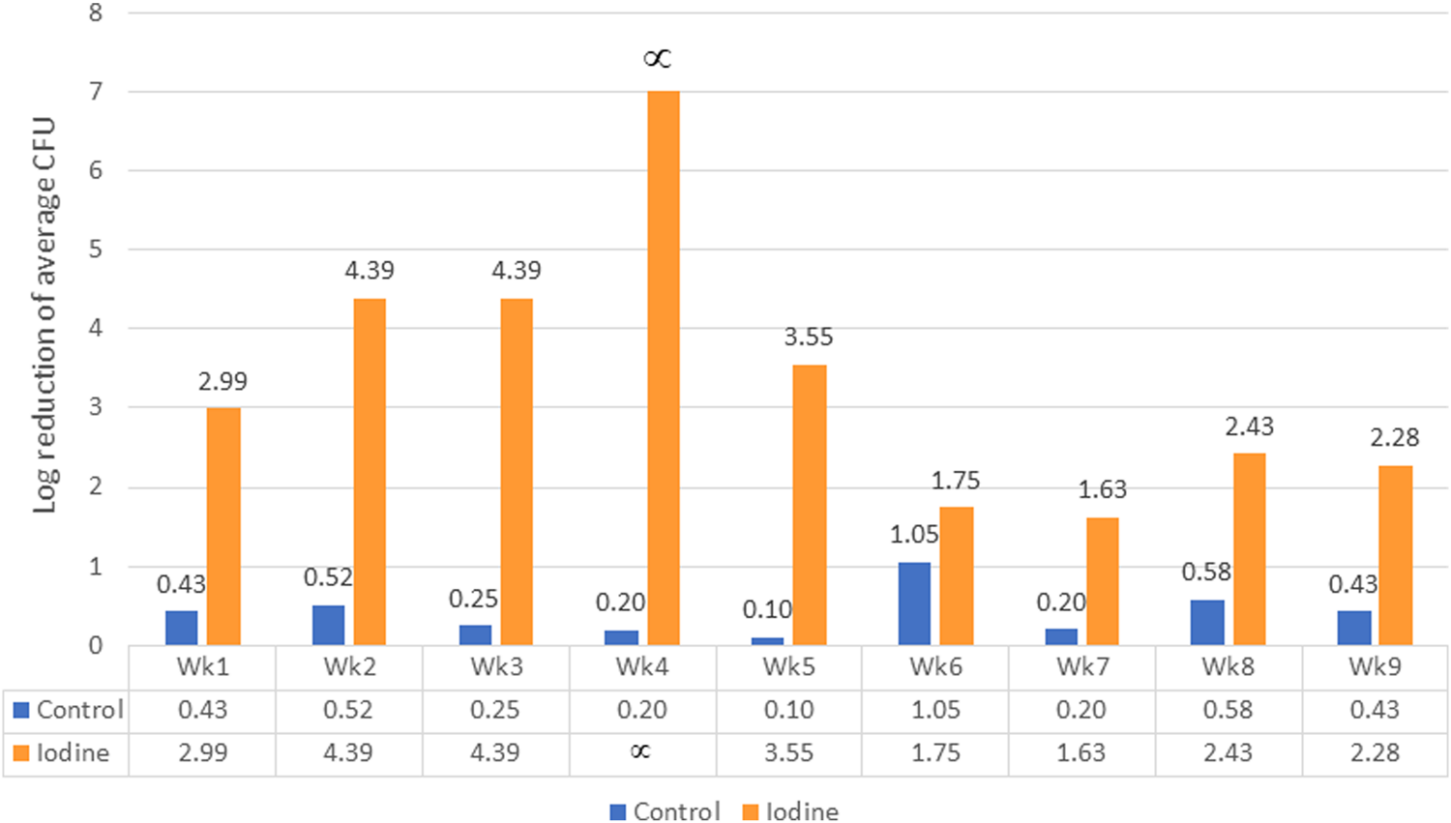
Log reduction of average CFU. The average CFU in the control and iodine group at each time point was calculated into Log reduction by comparing it to the average CFU of the baseline week. The blue bars indicate Log reduction of average CFU in the control group. The orange bars indicate Log reduction of average CFU in the iodine group.

The number of bacteria drastically decreased from the first week of continuous iodine treatment. The average CFUs/ml of bacteria recovered from the airotors line of the iodine group is 354 ± 541 CFUs/ml, significantly lower than the control group (18,591 ± 9208 CFU/ml). The average CFU/ml was transferred into log reduction to compare the decontamination efficacy to the sterility assurance level at 6 log reduction. The effectiveness of the iodine treatment was determined in a log reduction ranging from 1.63 to 4.39 log, except in the fourth week when the log reduction could not be calculated because no bacteria recovered from the sample (Fig. 3).

At week 9, the viability of biofilms in DUWLs was assessed by the amount of ATP. Biofilms in the iodine group had slightly lower ATP than the control group, though not statistically significant.

After eleven months of installation, the iodine concentration was measured to determine the potency of the cartridge. The average iodine concentration released in DUWLs procedural water was measured to be 3.6 ppm. This amount of iodine was still able to control bacterial contamination in the DUWLs, as demonstrated by the average bacterial CFU/mL (3125 ± 2499 CFU/ml) in the iodine group, which was significantly lower than the control group (59,250 ± 26,538 CFU/ml).

## Discussion

We found that continuous iodine treatment effectively controlled bacterial contamination in DUWLs from the beginning after installation. However, it is less likely to deprive biofilm viability as measured by the amount of ATP.

Continuous treatment is more effective than intermittent treatment in chemically controlling bacterial contamination in DUWLs [13]. Although both intermittent and continuous treatment hardly eliminated biofilms from the DUWLs, the continuous presence of disinfectant in the waterline could better slow the growth of biofilms and kill the planktonic form of bacteria in the output water. In contrast, intermittent treatment has a quick antimicrobial action only on the surface of biofilms. The regrowth of bacteria could be detected within a couple of days after the disinfecting cycle [4].

Several continuous treatments have been proven to effectively decontaminate DUWLs, such as plasma sterilization, ozonated water, electrolyte water system, or hypochlorous acids [10, 14, 17]. However, most of the system usually needs an automated machine to freshly prepare the disinfecting agent in real-time at the point of use [14]. Besides continuously providing disinfecting agents, the automated systems are effective because no staff compliance is needed; on the other hand, they can reduce human error. However, the automated system is costly and requires prompt maintenance.

Meanwhile, the Iodine cartridge water treatment is commercially available as an adapter cartridge that can be easily installed with an independent dental unit water reservoir bottle. It does not need an electrical supply, so it is easy to install. Thus, it requires less attention than the automated system. The longevity of iodine released from the cartridge is limited to the amount of water passed through the cartridge at ~240 l. Thus, it is necessary to track water input. It is recommended to check the iodine level after 11 months or as it approaches 240 l of water to ensure that the iodine output has not fallen below 0.5 ppm, the minimum effective concentration. Our result demonstrated that the iodine level in DUWLs, determined by iodide electrodes, is 3.6 ppm on average. This concentration was higher than the minimum effective level and still effectively reduced bacterial contamination in the DUWLs.

The maximum dosage of iodine with no side effects is 1000 mg/day for children and 2000 mg/day for adults. Procedural water for irrigating oral cavities in each dental treatment is less than 100 ml, and the patient may unintentionally swallow a small quantity of water [18]. The iodine level at 3.6 ppm can be inferred that 360 μg/day is the maximum dose the patient potentially swallows in one visit. It is less than all ranges of toxic doses, so this iodine level is safe for all ages [19].

However, the toxicity of iodinated water should be a concern in vulnerable patient groups such as iodine or seafood allergies, pregnancy, lactation, and some medical condition like autoimmune thyroid disease or having a history of chronic iodine deficiency [19]. In case of excessive iodine intake in vulnerable patient groups can cause alterations in thyroid function [20, 21]. Therefore, taking a medical history is essential to prevent adverse events. It is needed to inform recommendations about iodine safety doses for vulnerable patient groups. Only a trace amount of iodine is utilized in the cartridge system for DUWLs decontamination. Therefore, the residual iodine in the dental unit procedural water is much less than the concentration from recommended doses. So, it is acceptable for vulnerable patient groups to expose to such a low dose of iodine during dental treatment [10].

In this study, the viability of biofilms was determined by the amount of ATP. This indirect assessment method uses a bioluminescence assay to measure ATP. Living microorganisms store energy in the form of ATP. Therefore, the detection of ATP indirectly reveals the presence of microorganisms. The amount of ATP is directly proportional to the amount of light emitted and read out as RLU. Although RLU cannot be calculated to CFU, the higher RLU value reveals more viable biofilms in the sample.

One limitation of this study is that dental chair units in dental faculty are typically not in use daily, such as during weekends, national holidays, or examination week. The stagnant water during unused periods might affect the number of bacteria that recover from the waterlines. During the experimental period, there was a final examination on week 5. The change in the clinical course after the final examination might affect the utilization of the dental chair unit. This occurrence may explain the increase in bacteria in the later week. The lower number of bacteria in the control group on week 6 is an unexpected result. It might be due to the unpredictable nature of biofilm dispersion or a change in dental chair unit utilization on the day of sample collection.

## Conclusion

Continuously supplying iodine in DUWLs is an effective measure to control microbial contamination and biofilms in DUWLs.

## Data Availability

The authors confirm that the data supporting the findings of this study are available within the article. Raw data that support the findings are available from the corresponding author, upon reasonable request.
